# Orofacial features and pediatric dentistry in the long-term management of Infantile Pompe Disease children

**DOI:** 10.1186/s13023-020-01615-1

**Published:** 2020-11-23

**Authors:** Angela Galeotti, Sara De Rosa, Roberto Uomo, Carlo Dionisi-Vici, Federica Deodato, Roberta Taurisano, Giorgia Olivieri, Paola Festa

**Affiliations:** 1 Division of Dentistry, Bambino Gesù Children’s Research Hospital IRCCS, Rome, Italy; 2 Division of Metabolism, Bambino Gesù Children’s Research Hospital IRCCS, Rome, Italy; 3 Dentistry Unit, Department of Pediatric Surgery, Bambino Gesù Children’s Research Hospital, Viale Baldelli 41, 00146 Rome, Italy

**Keywords:** Glycogen storage disease type II, Pompe disease, Pediatric dentistry, Oral signs, Craniofacial growth, Non-invasive ventilation, Orthodontics, Oral functions

## Abstract

**Background:**

Glycogen storage disease type II (GSDII) or Pompe disease is a rare autosomal recessive metabolic disorder that leads to intracellular glycogen storage in many tissues, mainly in skeletal muscle, heart and liver. Facial muscle weakness and altered craniofacial growth are very common in Pompe disease children. In this paper we describe the orofacial features in two children affected by GSDII and illustrate a multidisciplinary approach that involved enzyme replace therapy, non-invasive ventilation (NIV) and pediatric dentistry with 5-year follow-up.

**Results:**

Two Infantile Pompe Disease children were examined by a pediatric dentist at the age of 4 and 5 years old respectively. The orofacial examination showed typical facies with similar features: hypotonia of facial and tongue muscles, lip incompetence, narrow palate with reduction in transversal dimension of the upper dental arch, macroglossia, low position of the tip of the tongue, concave profile, Class III malocclusion with hypoplasia of maxillary-malar area and mandibular prognathism. Myofunctional therapy and orthodontic treatment consisted in oral muscle exercises associated to intraoral and extraoral orthodontic devices. NIV facial mask was substituted with a nasal pillow mask in order to avoid external pressure on the mid-face which negatively influences craniofacial growth.

**Conclusions:**

This paper evidences that the pediatric dentist plays an important role in craniofacial growth control, oral function rehabilitation and, therefore, in the improvement of the quality of life of Pompe children and their families. Therefore an early pediatric dental evalutation should be included in the multidisciplinary management of children suffering from Infantile Pompe Disease.

## Background

Glycogen storage disease type II (GSDII) or Pompe Disease (PD) is a rare autosomal recessive metabolic disorder caused by a deficiency of lysosomal enzyme, acid *α*-glucosidase (*GAA*), leading to intracellular glycogen storage in many tissues, mainly in skeletal muscle, heart and liver [1]. The diagnosis of PD is based on the measurement of GAA enzyme activity in blood (leukocytes or dried blood spot) or tissues (fibroblasts or muscle) and further confirmation of gene mutations by molecular analysis of *GAA* gene, which is located on chromosome 17q23 [2]. PD is usually classified into Infantile-Onset Pompe Disease (IOPD) and Late-Onset Pompe Disease (LOPD) based on whether clinical symptoms develop prior to or beyond 1 year of age, respectively. IOPD is further subdivided into either classic or non-classic forms: classic IOPD is the most severe form presenting within the first few months of life with cardiomyopathy. The non-classic IOPD usually appears within the first year of life with motor delays and/or slowly progressive muscle weakness, the cardiomyopathy can be present, but less severe than classic IOPD [2]. The clinical phenotype varies according to the age of patients and to the rate of disease progression. Infants with classic IOPD typically present during the first few weeks of life a combination of hypotonia, progressive weakness, hypertrophic cardiomyopathy, respiratory insufficiency. Then hepatomegaly, macroglossia, failure to thrive are often developed. This typical clinical presentation leads immediately to diagnosis. Untreated patients with IOPD follow a rapidly progressive and fatal course within first years of life [3].

On the other hand, the diagnosis of non-classic IOPD and LOPD can be more difficult: these patients generally present various degrees of slowly progressive limb-girdle weakness simulating the muscular dystrophies and respiratory difficulties without significant cardiomyopathy [4].

Since 2006, the Enzyme Replacement Therapy (ERT) with recombinant GAA (rhGAA) has significantly improved the overall prognosis. In patients with IOPD, ERT significantly increased long-term survival and determine sustained improvement of cardiac, respiratory and gross motor functions [5].

More than a decade after the introduction of ERT, data on follow-up in IOPD patients showed that long-term survivors encounter many problems due to residual muscle weakness, speech disorder, hearing loss, arrhythmia [6]. A recent retrospective observational study analyzed the long-term effectiveness of ERT on a large sample of IOPD children [7]. Facial muscle weakness, hyper-nasal speech, ptosis, and hearing loss are also very common in long-term follow-up of patients with IOPD diagnosed by newborn screening and treated since birth [8].

To date only few reports are available on orofacial manifestations in PD children [9–11] and no studies reported the involvement of pediatric dentistry in the long-term management of children affected by GSDII. Thus, the purpose of this paper is to report the orofacial features and illustrate the contribution of the pediatric dentist and the orthodontist in the improvement of oral functions in two children affected by IOPD.

## Methods

This paper reports a multidisciplinary diagnosis and management of the orofacial manifestation in Infantile Pompe Disease children with long-term follow-up. Two Infantile Pompe Disease children under treatment at the Division of Metabolism of Bambino Gesù Children’s Hospital (Rome, Italy) were referred to Dentistry Unit for oral evaluation. Patients were examined by a pediatric dentist also specialist in orthodontics, who collected a complete anamnesis on the history of the patients and general health condition including breathing, feeding, swallowing and masticatory abilities. Then an extraoral and intraoral examination was performed in order to have a complete dental and orthodontic diagnosis. At the second dental examination, a full orthodontic records collection was performed: extraoral photographs, intraoral photographs and alginate impressions of upper and lower dental arches to form orthodontic study.

A detailed description of the diagnostic and therapeutic path of the two patients follows.

## Results

### Case 1

A 3-month-old boy was initially admitted at the Bambino Gesù Children’s Research Hospital (Rome, Italy) for severe hypotonia, splenomegaly, growth arrest and cardiomegaly.

He was the second-born of non-consanguineous healthy parents at 37 weeks from an uneventful pregnancy, his birth weight was 3200 g, head circumference was 41 cm and length was 50 cm, APGAR index was 6/8.

He was diagnosed with classic IOPD based on the measurement of GAA enzyme activity in leukocytes (0.26 nmol/mg prot/h n.v. 10–40) and further confirmation of gene mutations by molecular analysis of *GAA* gene (homozygous c.784G > A). The clinical manifestations of the patient were severe hypertrophic cardiomyopathy, hepatomegaly and muscle hypotonia. He was treated with intravenous rhGAA at standard dose since he was 3 months and with non-invasive nocturnal ventilation (NIV) by face mask from the age of two due to progressive chronic respiratory failure. Since the age of 4 years of age ERT dose was increased up to 40 mg/kg every week.

At the same age, he was referred to the Dentistry Unit of Bambino Gesù Children’s Research Hospital (Rome, Italy) for oral evaluation.

**Fig. 1:**
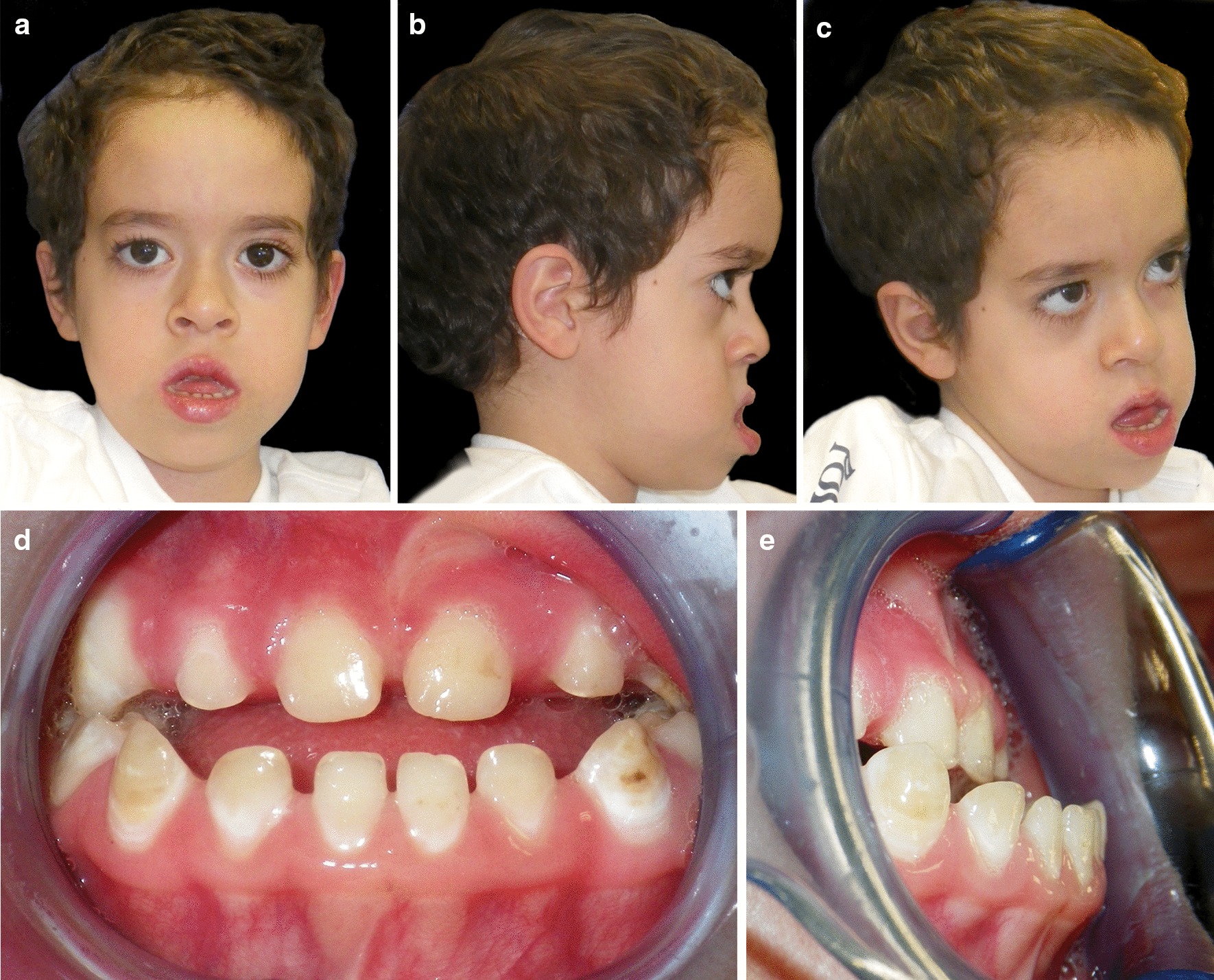
*Case 1*: **a**–**c** Facial extraoral photographs before dental treatment. **d**, **e** Intraoral views before dental treatment

Extraoral examination revealed long face, concave profile, oral breathing with lip incompetence, hypoplasia of maxillary-malar area and mandibular prognathism (Fig. 1a–c).

Intraoral examination revealed a primary dentition with anterior cross-bite, narrow palate with a reduction in the transversal dimension of the upper dental arch, Class III malocclusion, macroglossia, low position of the tip of the tongue and impaired eruption of the molars (Fig. 1d, e). The enamel of the deciduous teeth showed dark spots of hypomineralization and two dental decays were diagnosed.

The patient had serious feeding problems and assumed smoothie foods. The child’s parents complained also from saliva and food retention in the mouth due to difficulty in swallowing and chewing for facial and tongue muscle hypotonia. He also had orobuccal dyspraxia and gastroesophageal reflux. Furthermore, the parents reported that the air inflated by the NIV leaked out of the mouth because of lip incompetence and the sleep polygraphy evidenced an average saturation of 94%.

The patient was treated with a multidisciplinary approach including ERT, orofacial myofunctional therapy, oral and orthodontic treatments.

At the Dentistry Unit the child received instructions on oral hygiene care at home and underwent oral hygiene treatment sessions and conservative therapies for occlusal caries of teeth 8.5 and 7.5. Then, periodic topical fluoride treatments for prevention of decays were executed.

The orofacial myofunctional therapy included oral exercises to improve facial muscle weekness, swallowing and chewing.

Due to the general clinical conditions and difficulties in feeding the use of fixed orthodontic was avoided and the child started a treatment with high-pull extraoral chin cup traction (Micerium S.p.A. 16036 Avegno, Ge, Italy) for about 9–11 h/day. In order to reinforce oral muscles, masticatory exercises were prescribed during chin cup use.

**Fig. 2:**
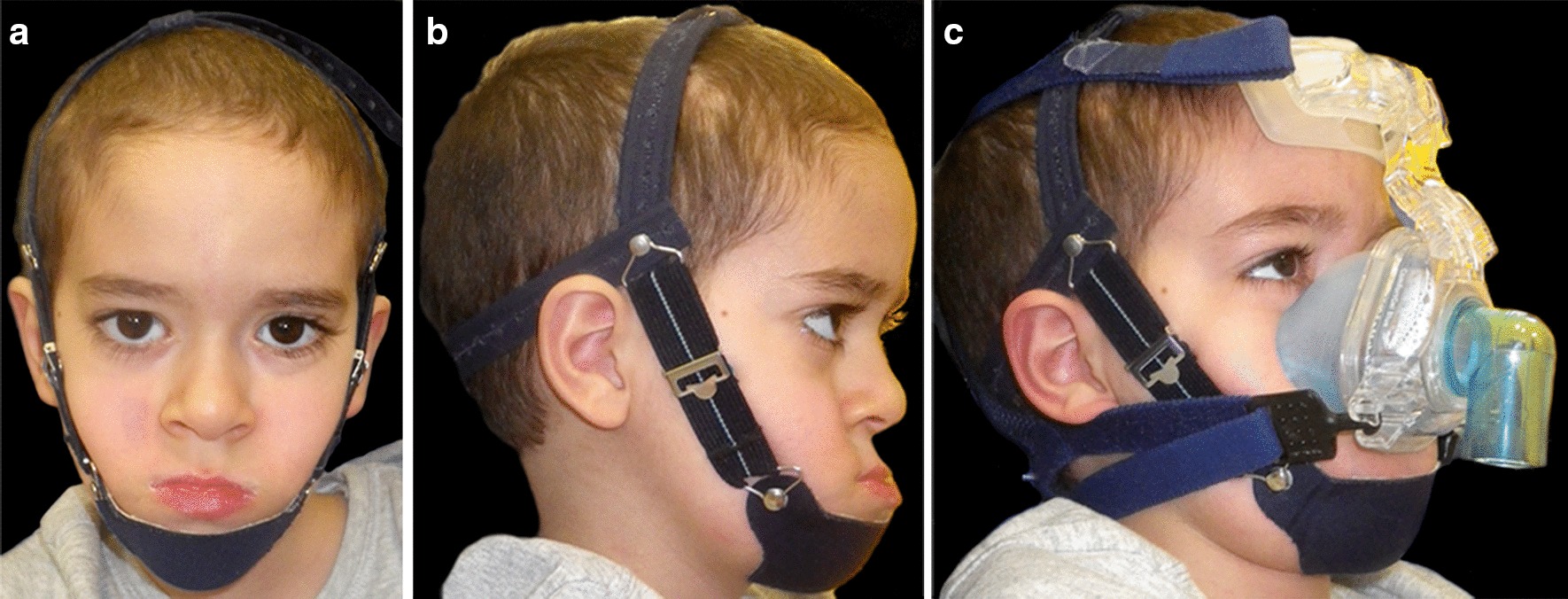
*Case 1:* **a**, **b** Facial extraoral photographs with chin cup. **c** The use of nasal face mask for NIV associated with chin cup

The use of overnight chin cup had several objectives: to maintain lip sealing, to prevent air leaks from the mouth during NIV use at night and to control mandibular sagittal and vertical growth. A polygraphic exam performed with chin cup associated with NIV mask reported an increase of average saturation at 97%. Figure 2 shows the use of chin cup, face mask for NIV and their association.

After only 3 months, the mastication significantly improved and the child started to chew food in pieces such as bread, pizza or meat. Due to chewing exercises the passive eruption of posterior teeth was favored, the facial muscle tone and lip competence were enhanced.

**Fig. 3:**
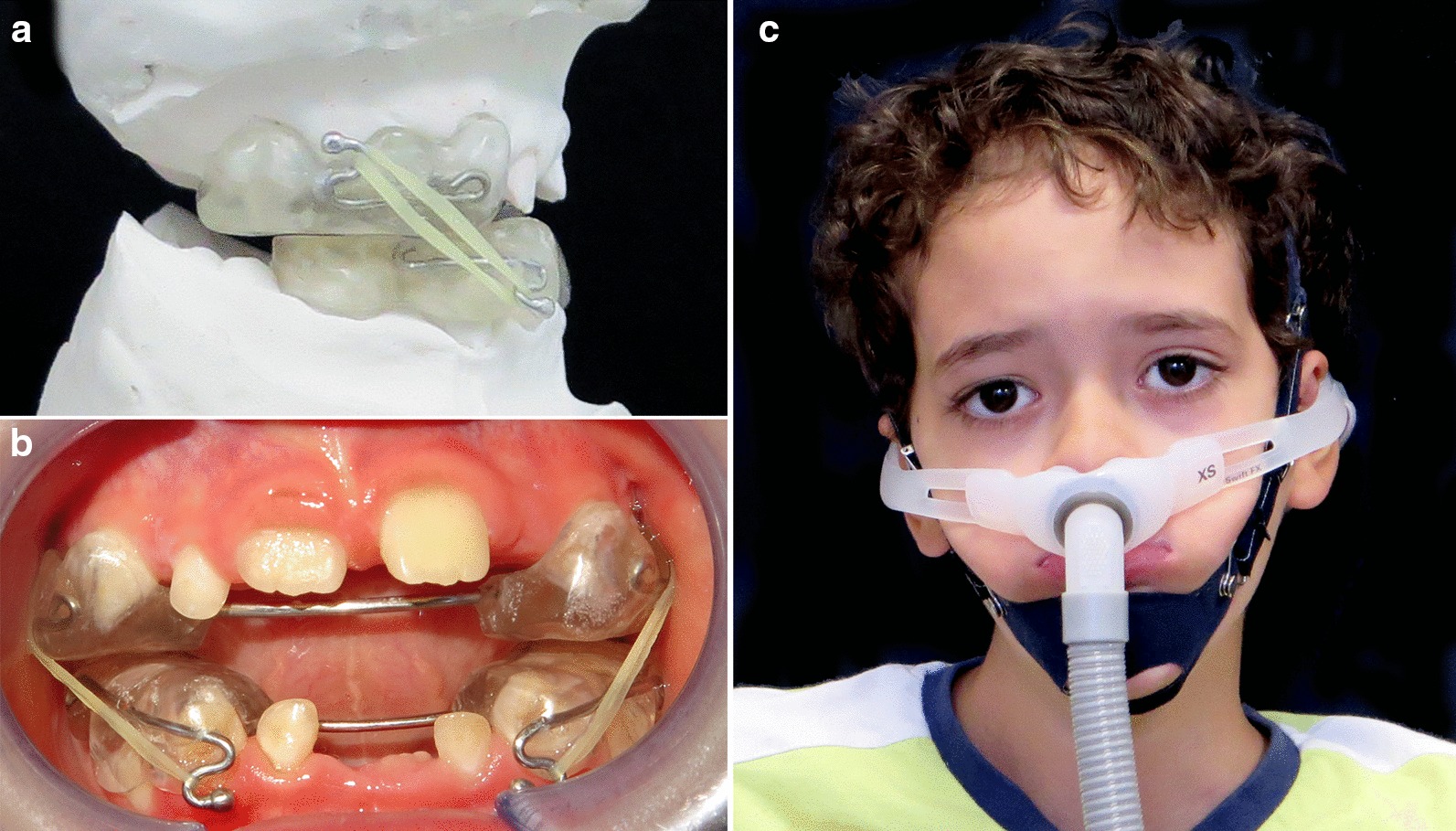
*Case 1*. **a** Lateral view of Class III splints with elastics on the orthodontic casts. **b** Introral view of Class III splints with elastics. **c** Nasal pillow mask for NIV associated with chin cup

At the age of seven, the orthodontic therapy included removable intraoral Class III splints with elastics (Fig. 3a, b) [12] with the aim of improving the skeletal sagittal discrepancy and the dental occlusion. Meanwhile, the NIV face mask was replaced with nasal pillows, a lightest and least invasive mask, to avoid external pressure on the mid-face (Fig. 3c) and possible interferences with the orthodontic device.

**Fig. 4:**
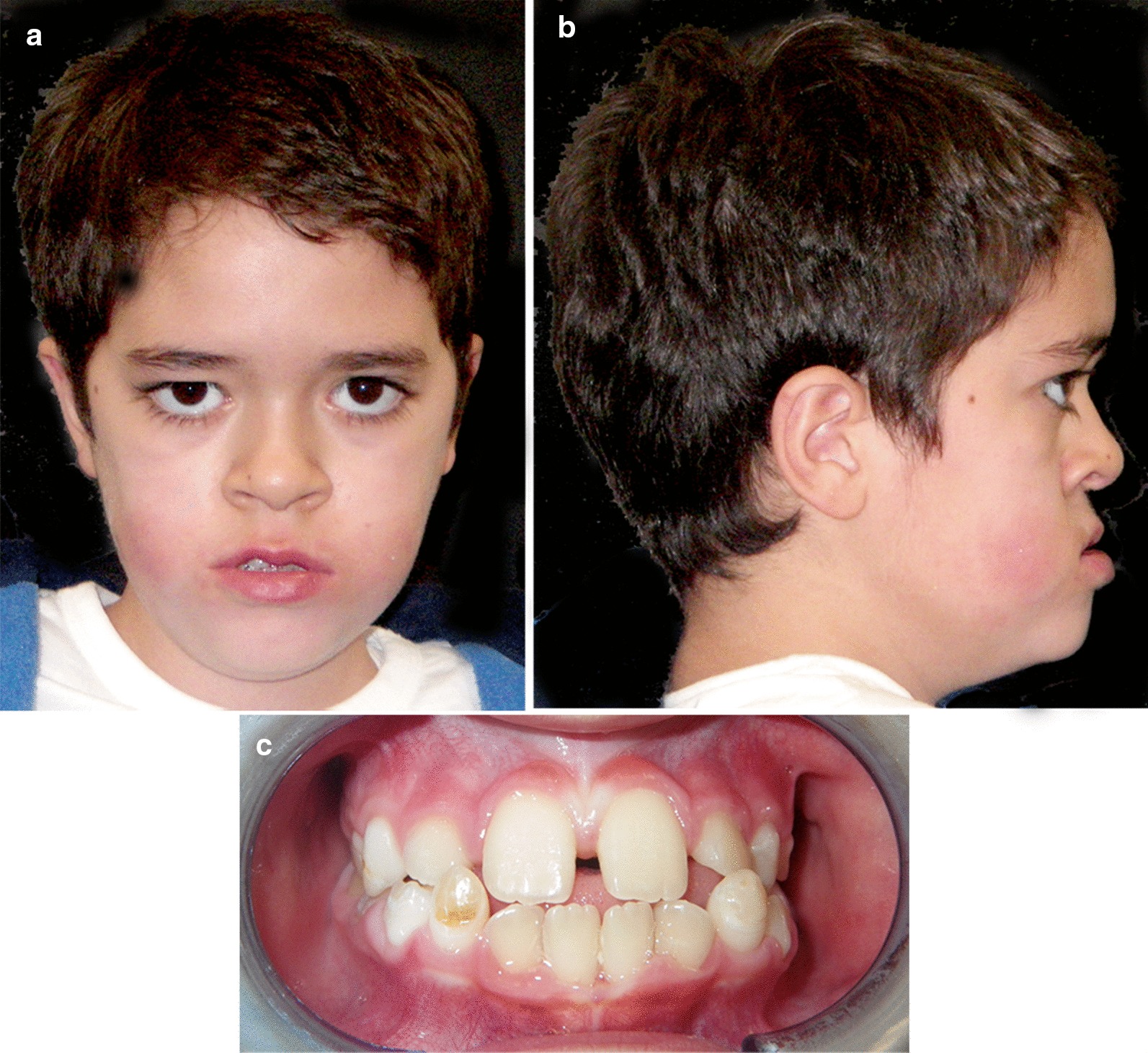
*Case 1.* **a**, **b** Facial extraoral photographs at the age of nine. **c** Intraoral view at the age of 9 years old

At the age of nine, the facial muscle tone and lip competence was significantly improved (Fig. 4a, b) and the dental occlusion showed a good compensation (Fig. 4c). The parents reported an improvement in night breathing, masticatory function and feeding ability that resulted in a great improvement in patient’s quality of life.

### Case 2

A 2-year-old boy was initially admitted at Bambino Gesù Children’s Research Hospital (Rome, Italy) for motor delay.

He was the first-born of non-consanguineous healthy parents at 40 weeks from an uneventful pregnancy, his birth weight was 4000 g and length was 50 cm.

He was diagnosed with non-classic IOPD. The diagnosis was based on the measurement of GAA enzyme activity in leukocytes (0.1 nmol/mg prot/h n.v. 10–40) and further confirmation of gene mutations by molecular analysis of *GAA* gene (homozygous c.1064 T > C). The clinical manifestations of the patient were mainly muscle hypotonia and chronic respiratory failure. He was treated with intravenous rhGAA at standard dose and NIV due to chronic respiratory failure.

At 5 years of age, ERT dose was increased up to 30 mg/kg every week and the patient was referred to the Dentistry Unit of Bambino Gesù Children’s Research Hospital for evaluation.

**Fig. 5:**
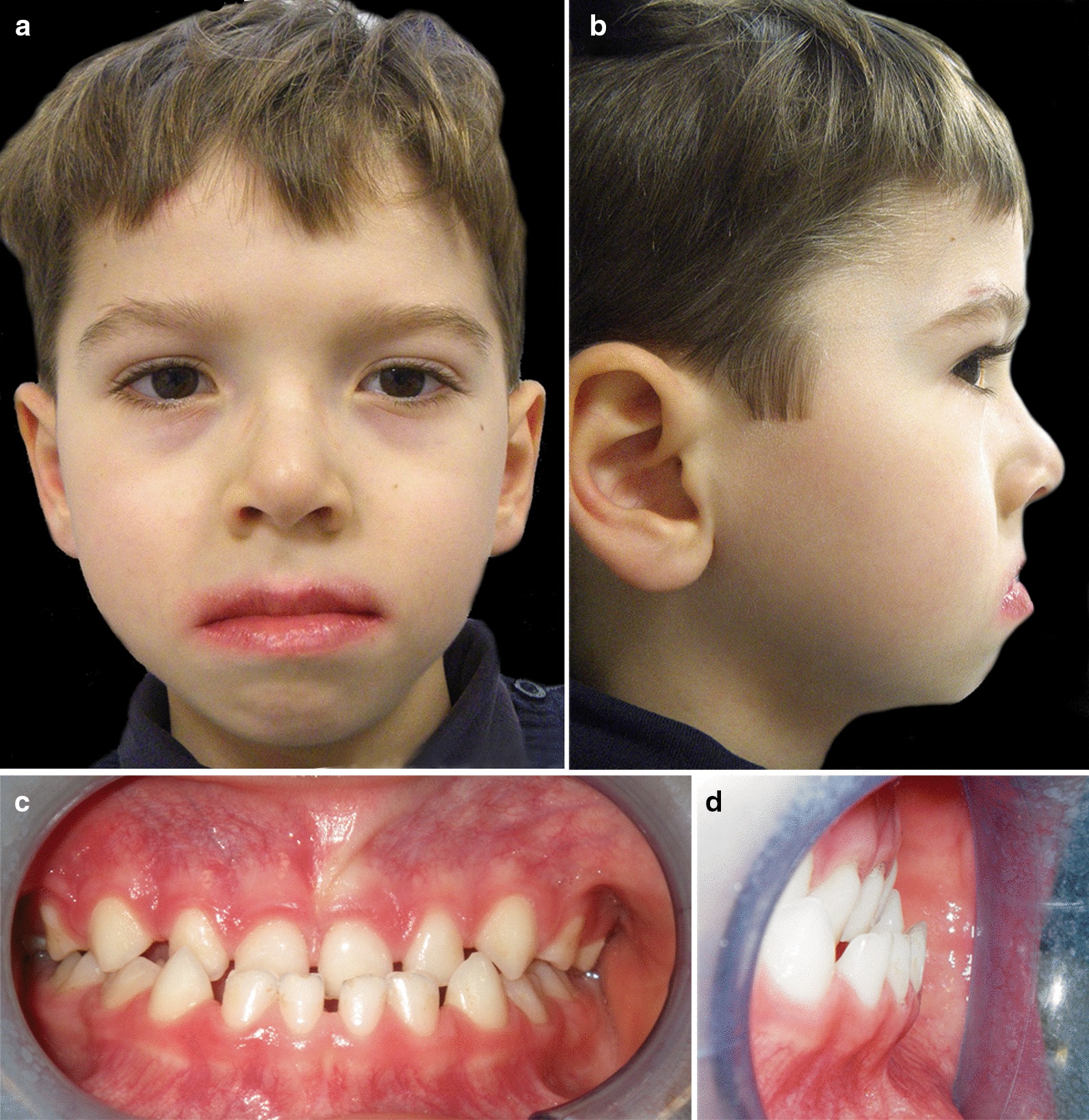
*Case 2*: **a**, **b** Facial extraoral photographs before dental treatment. **c**, **d** Intraoral views before dental treatment

Extraoral examination showed oral breathing, concave profile, hypoplasia of maxillary-malar area, mandibular prognathism, long face, lip incompetence, redness and dry skin around the lips (Fig. 5a, b).

Intraoral examination (Fig. 5c, 2d) revealed a primary dentition with anterior cross-bite, narrow palate with a reduction in the transversal dimension of the upper dental arch, Class III malocclusion, macroglossia, low position of the tip of the tongue and good oral hygiene.

The child was treated by a multidisciplinary approach including ERT, orofacial myofunctional therapy and orthodontic treatment.

At the Dentistry Unit the child and his parents were instructed on oral hygiene care at home and referred to a speech therapist to introduce an orofacial myofunctional therapy in order to improve facial muscle weakness, swallowing and chewing.

The patient started an orthodontic treatment with removable intraoral Class III splints with elastics [12] with the indication to progressively increase the daytime use based on patient’s tolerance. The patient showed a good compliance with the orthodontic device and the parents reported a significant improvement of masticatory function and lip sealing.

**Fig. 6:**
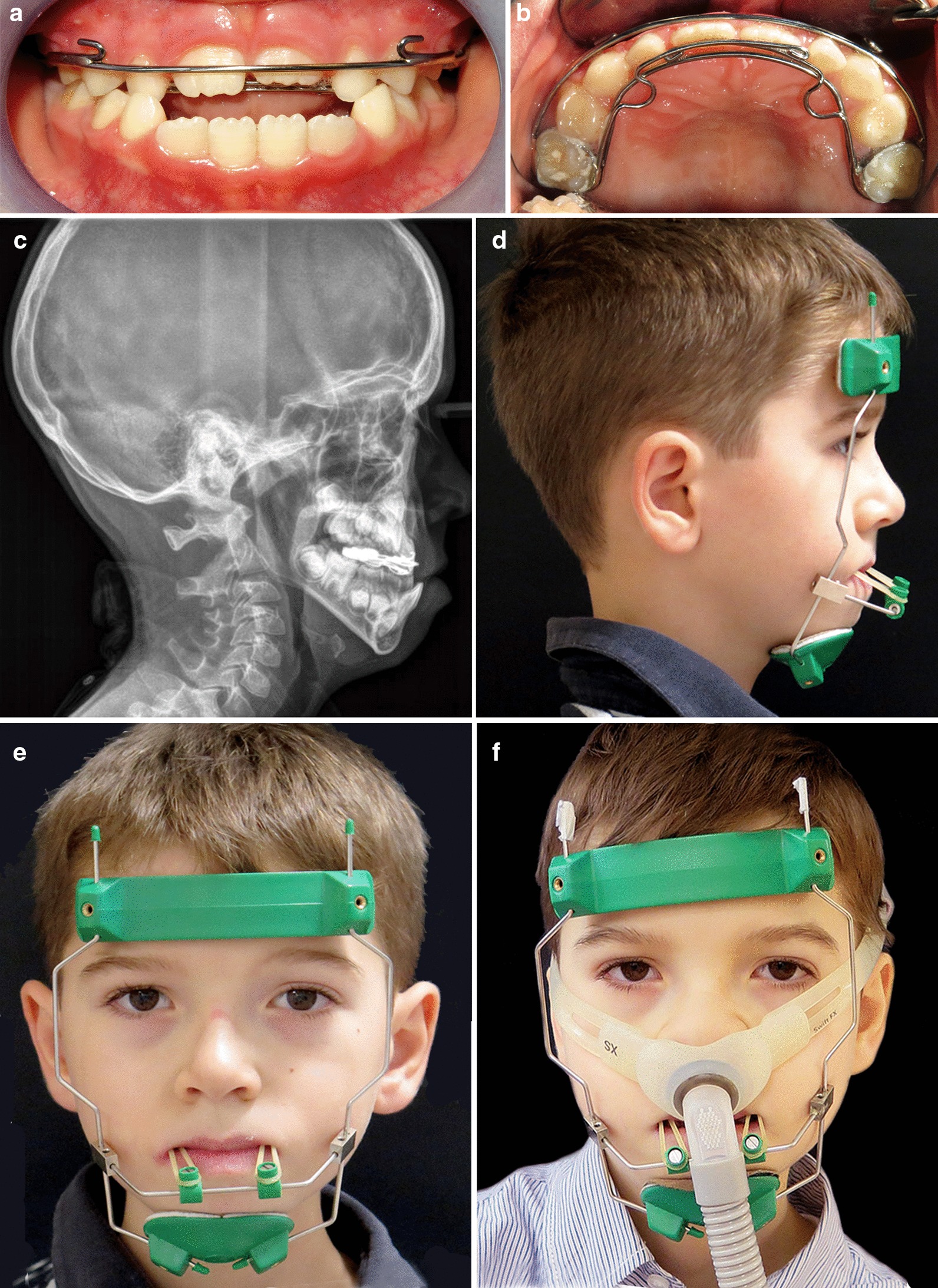
*Case 2.* **a**, **b** Intraoral frontal view and upper occlusal view with double arch in situ. **c** Lateral cephalogram at the age of 7 years old. **d**, **e** Extraoral orthodontic facemask in frontal and profile views. **f** Nasal pillows associated with extraoral orthodontic facemask

After about 2 years, a fixed orthodontic treatment was started: it comprised a double arch soldered to bands on the upper first permanent molars with hooks in the maxillary canine region (Fig. 6a, b). A lateral cephalogram was performed which showed evidenced a severe discrepancy of Class III skeletal malocclusion (Fig. 6c). The use of an extraoral orthodontic facemask (Fig. 6d, e) was associated in order to procline upper incisors and to improve Class III sagittal discrepancy. At the same time the NIV facial mask was replaced with nasal pillow mask to avoid external pressure on the mid-face and to avoid interferences with the extraoral orthodontic device (Fig. 6f).

The patient had good compliance with the orthodontic devices and the parents reported a great improvement in nighttime breathing while using the orthodontic facemask.

**Fig. 7:**
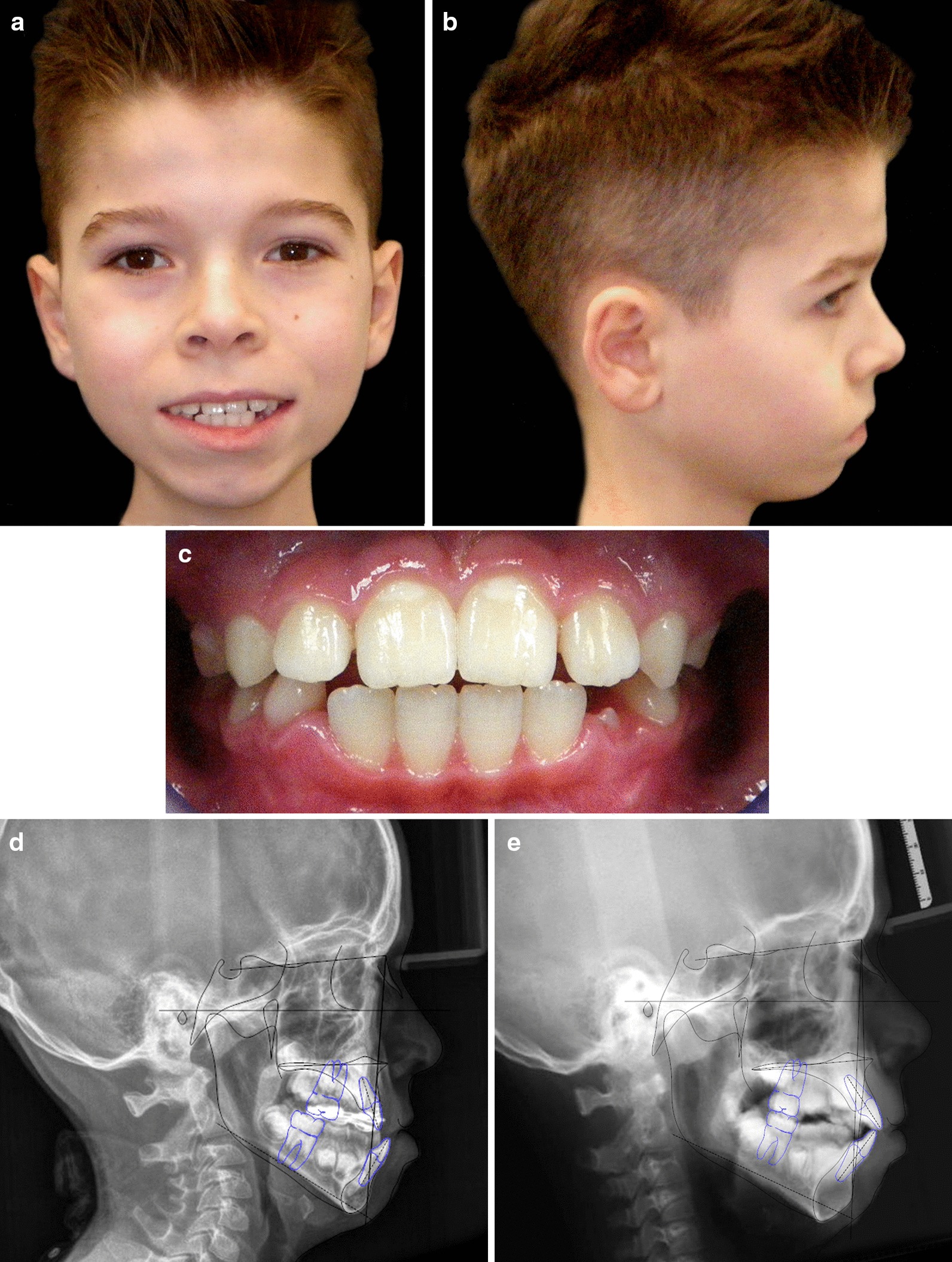
*Case 2.* **a**, **b** Facial extraoral photographs at the age of ten. **c** Intraoral view at the age of 10 years old. **d**, **e** Pre and post treatment lateral cephalograms with tracing

At the age of ten, the extraoral view showed good labial competence, significant profile improvement (Fig. 7a, b) and the occlusion had good compensation (Fig. 7c). The comparison between pre and post treatment lateral cephalograms showed a significant advancement of the upper maxilla (Fig. 7d, e). Facial muscle tone and lip competence were enhanced and feeding ability significantly improved, resulting in a better quality of life for patient and their parents.

## Discussion

The management of patients with Pompe Disease requires a coordinated approach that involves different specialists [13].

The prevalence of weakness involving orofacial muscle is high in Pompe children [8, 13] and some orofacial features reported in this study have been previously described in PD children, such as lip incompetence [14, 15] and low position of the tongue with macroglossia [15].

Both children described in this paper presented craniofacial anomalies that may be addicted to facial muscle weakness associated with mouth breathing: an increase of anterior lower facial height, mid-face hypoplasia and palatal constriction. As reported previously [16], the long-term mouth breathing plays a significant role in influencing craniofacial growth and determining an increase of the anterior lower facial height. Moreover the absence of physiological nasal breathing in mouth-breathers induces a decreased nose prominence and nasal width dimensions compared to normal children [17] and may hesitate in maxillary-malar area hypoplasia.

The lower position of the tongue may influence the craniofacial growth because it results in a lack of internal pressure leading to a reduction of transversal growth of the upper arch with the development of lateral and posterior crossbite [16, 17]. This mechanism contributes to the transverse maxillary deficit but also to the vertical increase of the mandibular growth evidenced in these patients. Moreover, it was reported that the tip of the tongue is in a more anterior position in the Class III malocclusion patients [18]. We hypothesize that the anterior tongue position may stimulate a sagittal mandibular growth promoting the development of mandibular prognathism and concave profile in PD children. Therefore, the early treatment of orofacial functions assumes an important role in the management of Pompe patients in order to limit the onset of severe deficit in swallowing, speech, chewing and breathing, as suggested in a previous study [19].

On the other hand, Class III malocclusion is associated with a reduced masticatory efficiency [20], so the use of orthodontic devices to correct Class III skeletal malocclusion may determine a significant improvement in mastication and feeding ability that is often reduced in Pompe Disease children due to muscle weakness.

The deficit of maxillo-malar area in the described PD children is another important evidence. It is concevaible that it is due to negative effects of long-term use of nasal non-invasive ventilation (NIV) on the facial growth even because no familiarity was reported for Class III skeletal malocclusion in both patients. As previously suggested, NIV may limit the sagittal upper maxilla growth and have negative effects on the development of facial bones [21]. A global facial flattening concerned malar area and maxilla, a concave profile and a maxillary retrusion was reported in 68% of the patients by Fauroux et al. [22] that analyzed 40 children on Non-invasive Positive-Pressure Ventilation (NPPV) because of obstructive sleep apnea. Furthermore, Li et al. [23] reported that long-term application of the tight-fitting headgear and face mask for at home nasal continuous positive airway pressure resulted in the retardation of facial skeletal development with a mid-face hypoplasia in a growing child. We suppose the hypoplasia of maxilla-malar region hesitates in a further reduction of upper airway space that also negatively influence the breathing capacity in PD children and their response to Continuous Positive Airway Pressure (CPAP) therapy. For the above mentioned reasons, we managed both patients in two ways: orthodontic extraoral forces consisted of chin cup and facial mask to oppose the negative effect on facial growth and the substitution of the NIV mask with nasal pillow. The orthodontic treatment compliance was successful for both patient and their families also because they were encouraged by continuous improvements in the oral functions.

The novelty of this paper is the accurate description of craniofacial features and the involvement of pediatric dentistry in the multidisciplinary management of PD children with long-term follow-up. Our experience evidences that the pediatric dentist plays an important role, not yet reported: in a recent paper about evidence-based guidelines in Pompe Disease treatment, the dentist is not even mentioned among the healthcare providers involved [14].

Further investigations are needed for evaluate the effects of the orthodontic approach in the management of a large sample of PD children.

## Conclusions

The development of orofacial anomalies in Pompe children examined in this study may be related to both muscle weakness and use of NIV with face mask that negatively affect the craniofacial growth. These evidences suggest that an early pediatric dental evaluation should be included in the multidisciplinary management of children suffering from infantile PD. The orthodontic treatment results evidence that this approach is useful in controlling craniofacial growth and improving oral functions, feeding ability and, therefore, the quality of life of children and their families.

## Data Availability

Not applicable.

## Abbreviations

ERT: Enzyme replacement therapy
*GAA*: Acid *α*-glucosidase
GSDII: Glycogen storage disease type II
IOPD: Infantile-Onset Pompe Disease
LOPD: Late-Onset Pompe Disease
NIV: Non-invasive ventilation
rhGAA: Recombinant acid *α*-glucosidase
PD: Pompe Disease

## Data sharing and supplemental materials

Data sharing not applicable to this article and no data sheets were generated or analyzed during the current study.
